# Sensitivity to BST-2 restriction correlates with Orthobunyavirus host range

**DOI:** 10.1016/j.virol.2017.06.017

**Published:** 2017-09

**Authors:** Mariana Varela, Ilaria M. Piras, Catrina Mullan, Xiaohong Shi, Natasha L. Tilston-Lunel, Rute Maria Pinto, Aislynn Taggart, Stephen R. Welch, Stuart J.D. Neil, Felix Kreher, Richard M. Elliott, Massimo Palmarini

**Affiliations:** a MRC-University of Glasgow Centre for Virus Research, 464 Bearsden Road, Glasgow G61 1QH, Scotland, United Kingdom; b Department of Infectious Disease, King's College School of Medicine, Guy's Hospital, Great Maze Pond, London Bridge, SE1 9RT London, England, United Kingdom

## Abstract

Orthobunyaviruses include several recently emerging viruses of significant medical and veterinary importance. There is currently very limited understanding on what determines the host species range of these pathogens. In this study we discovered that BST-2/tetherin restricts orthobunyavirus replication in a host-specific manner. We show that viruses with human tropism (Oropouche virus and La Crosse virus) are restricted by sheep BST-2 but not by the human orthologue, while viruses with ruminant tropism (Schmallenberg virus and others) are restricted by human BST-2 but not by the sheep orthologue. We also show that BST-2 blocks orthobunyaviruses replication by reducing the amount of envelope glycoprotein into viral particles egressing from infected cells. This is the first study identifying a restriction factor that correlates with species susceptibility to orthobunyavirus infection. This work provides insight to help us dissect the adaptive changes that bunyaviruses require to cross the species barrier and emerge into new species.

## Highlights

• BST-2 is a determinant of orthobuynyavirus host range.
• BST-2 restricts orthobunyavirus replication.
• BST-2 reduces the incorporation of envelope glycoprotein into virions.

## 1 Introduction

The increase in travel and commercial trade over the last two decades, in addition to climate and ecological changes, have facilitated the emergence of a variety of pathogenic viruses (Karesh et al., 2012). Several recently emerging viruses belong to the Bunyavirales order (classified until recently simply as the *Bunyaviridae* family), a large group of enveloped RNA viruses that comprise more than 350 named virus isolates divided among nine virus families and thirteen genera, including some which cause important diseases in humans, livestock and crops (Adams et al., 2017, Ergonul, 2012, McMullan et al., 2012, Yu et al., 2011). Within the *Peribunyaviridae*, the genus *Orthobunyavirus* includes more than 170 named viruses some of which are of medical and veterinary importance (Elliott, 2014). The orthobunyavirus genome comprises three segments referred to as large (L), medium (M) and small (S). The large segment encodes for the viral polymerase. The M segment encodes for the viral glycoproteins Gn-NSm-Gc that are synthesized as a poplyprotein precursor that is later proteolitically cleaved. The S segment encodes for the viral nucleocapsid and the non-structural protein NSs in an overlapping reading frame (Eifan et al., 2013).

Orthobunyaviruses like Oropouche virus (OROV), La Crosse (LACV) virus and Ngari virus can be the cause of febrile illnesses, encephalitis or hemorrhagic fevers in humans (Bowen et al., 2001, Pinheiro et al., 1981). On the other hand, orthobunyaviruses such as Akabane virus (AKAV), Sathuperi virus (SATV) and the recently emerged Schmallenberg virus (SBV) cause abortions and congenital malformations in ruminants (Calisher, 1996, Kurogi et al., 1975), while Cache Valley virus (CVV) causes disease both in ruminants and humans.

Important public health considerations need to be made when a new orthobunyavirus emerges. For example, SBV emerged in Germany in the summer of 2011 (Hoffmann et al., 2012) and spread very rapidly throughout Europe. On the bases of epidemiological data and the similarity of SBV to other orthobunyaviruses infecting ruminants, the risk of transmission of SBV to humans was considered low. Indeed, subsequent studies showed that no antibodies against this virus were detected in individuals in contact with infected animals (Ducomble et al., 2012, Reusken et al., 2012). Hence, with some notable exceptions (i.e. CVV), it appears that many of the medically or veterinary relevant orthobunyaviruses have a fairly strict host range as they cause disease either in humans or ruminants.

Virus tropism for a particular animal species is determined by many factors including, in some cases, innate immune responses. Vertebrates have evolved a variety of mechanisms to counteract exposure to pathogens. A key innate immune mechanism to fight virus infection is the type I interferon (IFN) and pro-inflammatory responses. Secretion of IFN by infected cells leads to autocrine and paracrine signaling resulting in the activation of hundreds of IFN-stimulated genes (ISGs) some of which have a direct or indirect antiviral effect (Nan et al., 2014, Yan and Chen, 2012). In turn, viruses have evolved mechanisms to counteract host restriction factors. Available evidence, mainly provided by studies on primate lentiviruses (Wain et al., 2007), suggests that viruses that have successfully established themselves in a given species are able to counteract, at least partially, the innate immune response of the host (Hrecka et al., 2011, Stremlau et al., 2004, Stremlau et al., 2006). Conversely, the same viruses cannot overcome the innate responses of non-susceptible species. Hence, sequence differences within ISGs orthologues could provide at least partial susceptibility or resistance to a given virus infection.

As most emerging human viruses are predicted to be zoonotic in origin (Mandl et al., 2014), it is important to assess the zoonotic potential of any newly discovered veterinary pathogen. Understanding the molecular mechanisms determining virus host range is key to gain insight into the rules that govern viral emergence. Cross-species transmission requires overcoming host-specific barriers that can be present at each stage of the viral replication cycle and can be specific for different animal species. Our understanding of the effect of host ISGs on bunyavirus host range, as well as the molecular adaptations required to overcome host genetic barriers is limited. In vitro, orthobunyaviruses grow extremely efficiently in a variety of cell lines derived from different species. Hence, the host range of these viruses does not seem to be due to species-specific cellular receptors or other factors absolutely required for virus replication. An ISG with a broad inhibitory activity against enveloped viruses is BST-2 (also known as tetherin, CD317, HM1.24) (Neil et al., 2008, Sauter, 2014, Van Damme et al., 2008). BST-2 is a type II membrane protein with an unusual topology consisting of a coiled-coiled ectodomain bound to the cell membrane by an N-terminal transmembrane domain and a C-terminal GPI anchor. The *BST2* gene is duplicated in ruminants: sheep and cows possess two *BST2* paralogs, *BST2A* and *BST2B*, both with antiviral properties although probably displaying different mechanisms of action (Murphy et al., 2014, Takeda et al., 2012). Ovine BST2B (oBST2B) displays particular features: it lacks predicted glycosylation sites and a carboxy terminal GPI anchor, resulting in a protein that is retained within the Golgi apparatus (Murphy et al., 2014). In this study, we investigated the role of BST-2 in determining orthobunyavirus host range. We found that BST-2 restricts orthobunyaviruses replication in a species-specific manner and thus likely contributes to determine the host range of this important group of viruses.

## 2 MATERIALS AND METHODS

### 2.1 Cell lines

HEK-293T and BSR cells were grown in Dulbecco's modified Eagle's medium (DMEM). BSR-T7/5 cells (provided by Karl Conzelmann) were grown in Glasgow modified Eagle's medium. Sheep choroid plexus cells (CPT-Tert) (Arnaud et al., 2010) were grown in Iscove's modified Dulbecco's medium. Human primary dermal fibroblasts were obtained from ATCC and cultured in fibroblast growth media 2 (Promo Cell). All cell lines were supplemented with 10% fetal bovine serum (FBS) and penicillin and streptomycin (p/s) and cultured at 37 °C in a 5% CO_2_ and 95% humidified atmosphere.

### 2.2 Antibodies

Antisera used in this study included a rabbit polyclonal antiserum against the SBV N protein (Proteintech) (Varela et al., 2013), a polyclonal antibody against SBV Gc protein (Gensript), a monoclonal antibody against Gc (Wernike et al., 2017) and a polyclonal antibody against whole OROV. Polyclonal antibody against the HA tag was obtained from Abcam. HIV p24 was obtained through the NIH AIDS Reagent Program (HIV-1 p24 Hybridoma (183-H12-5C)) (Chesebro et al., 1992). Antibody against γ-tubulin was obtained from Sigma. Fluorescent secondary antibody included AlexaFlour 488 or 594 goat anti rabbit (Life technologies).

### 2.3 Viruses

Wild type SBV was obtained by reverse genetics as previously described (Varela et al., 2013). AKAV was rescued by reverse genetics using the plasmids described below. The origin of SATV was described previously (Watret et al., 1985). OROV was supplied by Christian Drosten (Institute of Virology, Bonn Medical Centre, Bonn, Germany). LACV was supplied by Friedman Weber. The 6V633 strain of CVV was used.

### 2.4 Plasmids

Plasmids expressing the HA tagged version of human Bst-2 (pCR3.1-hBST2-HA), ovine Bst2-A and -B (pCIoBST2A-HA and pCIoBST2B-HA), an HIV-1 molecular clone deleted of VPU (HIVDΔVPU) and plasmid expressing VPU-tagged with the HA epitope have been previously described (Arnaud et al., 2010, McNatt et al., 2009, Neil et al., 2006, Neil et al., 2008). pCI (Promega) was used as an empty plasmid control. pUCSBVST7, pUCSBVMT7 and pUCSBVLT7 (Varela et al., 2013) encode the full-length antigenomic S, M and L SBV segments and were used to rescue SBV. TVT7R-SBVM-ren [-] encodes a chimeric antigenomic SBV M segment where the M protein coding region has been replaced by the *Renilla* luciferase gene. pTM1-SBV-N and pTM1-SBV-L express the N and L proteins of SBV under the control of the T7 promoter. pUCAKAVST7, pUCAKAVMT7 and pUCAKAVLT7 encode the full-length antigenomic S, M and L AKAV segments and were used to rescue AKAV by reverse genetics. Plasmids were synthesized commercially and derived from complete AKAV sequences available in GenBank (AB190458.1; AB100604.1; AB000851.1). The sequences of the chimeric BTS2 genes are presented as Supplemental information (Supplemental experimental procedures 1).

### 2.5 Concentration of virions

800–900 µl of infected supernatants were filtered, layered over 600 µl of cold 20% sucrose and virions were pelleted by centrifugation at 25,000*g* for 100 min. Virions were then resuspended in 20 µl of 1 x Laemmli buffer and heated at 95⁰C for 5 min followed by western blot analysis.

### 2.6 Recovery of intracellular infectious SBV

Cells were infected at a MOI of 0.001 in triplicate. 48 h post-infection supernatants were removed, cells washed with PBS with 2% FBS and scraped using 500 µl of PBS supplemented with 2% FBS followed by two cycles of freeze-thawing. Cell debris were then pelleted and supernatants titrated by limiting dilution. The experiment was performed three times independently.

### 2.7 Protease stripping assay

293-hBST2 or control cells were infected with SBV (MOI of 0.001) and 48 h post-infection supernatants were then collected, filtered and virions pelleted as described above. The remaining cells were washed twice with PBS before they were treated with either PBS, dilution buffer (10 mM Tris [pH 8.0], 1 mM CaCl2, and 150 mM NaCl) or subtilisin A (10 µg/ml dilution buffer) for 45 min at 37⁰C. The reaction was then stopped by the addition of 10% FBS DMEM and 5 mM of PMSF and supernatants were collected, filtered and virions pelleted as described above. Cells were lysed using 1X Laemmli buffer. Samples were analyzed by western blotting.

### 2.8 RNA interference

Lentiviral vectors were used to stably express hairpin RNAs against hBST2 or GFP as a control to knockdown BST-2 expression in human primary fibroblasts (Blondeau et al., 2013, Wilson et al., 2007). The relative levels of hBST-2 transcripts were estimated by quantitative RT-PCR sung the Brilliant III Ultra Fast qRT-PCR master mix and the following primers and probe: hBST-FW TGATGGCCCTAATGGCTTCC; hBST-RW AGACCTGGTTTTCTCTTCTCAGTCG; and hBST FAM-CCTCAAGCTCCTCCACTTTCTTTTGTCCTT-BBQ. Actin was used as a normalizing control. Reactions were cycled on a Stratagene Mx3005 qPCR System (Agilent Technologies) and data was analyzed with the Mx3000P software. The data is expressed as the Log10 relative reduction in expression compared to the cell line expressing a GFP shRNA.

### 2.9 Quantification of viral mRNA

293-hBST2 and control cells were infected (MOI of 0.001) for 1 h at 4⁰C to synchronize infection followed by 1.5 h at 37⁰C. Cells were then cultured for 1, 2 and 5 h when total RNA was extracted using the RNAeasy mini kit (Qiagen). Viral RNA was reverse transcribed using a SBV specific primer (5′ TTCGGCCCCAGGTGCAAATC 3′) with AccuScript HF reverse transcriptase following manufacturer's instructions. cDNA was used for qRT-PCR using the Brilliant III Ultra Fast QPCR master mix as indicated by the manufacturer. The following primers and probe were used: SBV-S-FW (TCAGATTGTCATGCCCCTTGC); SBV-S-RW (TTCGGC CCCAGG TGCAAATC); and SBV-S-FAM (TTAAGGGATGCACCTGGGCCGATGGC). Reactions were cycled on a Stratagene Mx3005 qPCR System (Agilent Technologies) and data was analyzed with the Mx3000P software.

### 2.10 Mini replicon assays

SBV mini replicon assays were performed in parallel in 293-hBST2 and 293T-control cells. Cells were transfected with 300 ng of TVT7R-SBVM-ren [-], 300 ng of pTM1-SBV-N, 150 ng of pTM1-SBV-L and 250 ng of pCMV-T7 plasmids using Transit-LT1 (Mirus Bio LLC) following the manufacturer's instructions. 24 h later, luciferase activity was measured using the Dual-Luciferase® Reporter Assays System (Promega). The experiment was performed four times, each time in triplicate.

### 2.11 Statistical analysis

Statistical analysis was performed using GraphPad Prism. All graphs show the averages and standard deviations.

## 3 Results

### 3.1 Human BST-2 restricts SBV replication

**Fig. 1:**
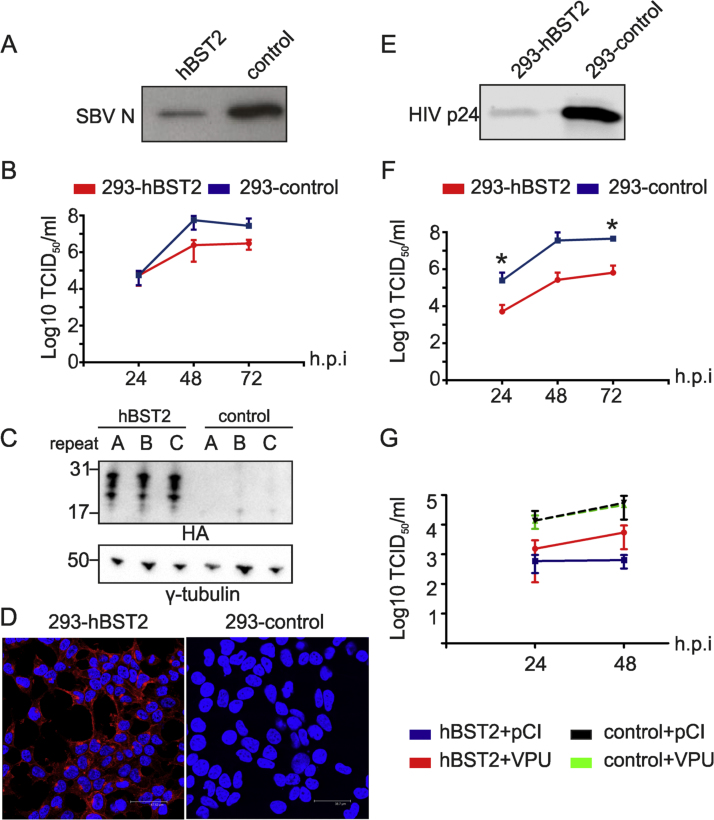
**Human BST-2 hampers SBV replication.** (A) Western blot analysis of SBV virions generated by rescue of SBV by reverse genetics in parallel to transient expression of hBST2 or an empty plasmid as a control. Virions were identified using antibodies towards the nucleocapsid (N) protein. (B) Representative experiment showing SBV growth kinetics in HEK 293T transiently transfected with 500 ng of an expression plasmid of hBST2 or an empty plasmid as a control before infection with SBV (top panel). (C) Western blot analysis (anti HA tag) confirming hBST2 expression in each of the three replicate wells (bottom panel). (D) 293-hBST2 and 293-control cells were analyzed by confocal microscopy using antibodies against the HA tag to detect hBST2 (red). No unspecific background staining was visible in control cells (not shown). (E) Western blot analysis of HIV-1 virions (capsid-p24) generated by transfection of 293-hBST2 and control cells with an HIV-1 molecular clone lacking the VPU gene. (F) SBV growth kinetics in 293-hBST2 cells. Results are expressed as the log_10_ TCID_50_ per ml of the average of three independent experiments, using two independent virus preparations (paired *t*-test). (G) Representative experiment showing SBV growth kinetics in 293-hBST2 and control cells transiently transfected with 500 ng of an expression plasmid of HIV-1 VPU or an empty plasmid (pCI) before infection with SBV (MOI 0.001). All the experiments displayed in this figure were done in triplicate and repeated at least three times. *P ≤ 0.05; ** P ≤ 0.01; ***P ≤ 0.001; ****P ≤ 0.0001.

Initially, in order to test the sensitivity of orthobunyaviruses to BST-2, we rescued SBV by plasmid transfection of BSR-T7/5 cells in the presence or absence of hBST2. Five days post-transfection, supernatants were collected, virus concentrated by centrifugation and pellets analyzed by western blotting (Fig. 1A). We found a reduction in the amount of SBV nucleocapsid protein (SBV N) in the supernatants of transfected cells suggesting less efficient rescue of SBV in the presence of hBST2. To confirm these results, HEK 293T cells were transiently transfected with a plasmid expressing the hBST2 gene or an empty plasmid as a control and 14 h post-transfection cells were infected with SBV at a low multiplicity of infection (MOI 0.001). Virus replication kinetics was monitored for 72 h. We found decreased SBV infectious titers in samples derived from cells transfected with hBST2 compared to control cells (Fig. 1B-C). We then developed HEK 293T cells stably expressing hBST2 (termed 293-hBST2) by transduction with retroviral vectors carrying hBST2 tagged with the HA epitope or an empty retroviral vector (termed 293-control) as a negative control. As shown in Fig. 1D, all cells expressed hBST2 and, as expected, this cell line was capable of restricting the release of HIV-1 lacking the accessory protein Vpu, a known BST-2 antagonist (Neil et al., 2008) (Fig. 1E). We then compared the SBV replication kinetics in 293-hBST2 and 293-control (Fig. 1F). We found a 10-fold reduction in the titers of SBV produced in 293-hBST2 cells compared to those obtained in 293-control. We obtained the same results using another independently established HEK 293T cell line stably expressing huBST2 (not shown). Importantly, the restriction of SBV in 293-hBST2 cells could be partially overcome by transient transfection of a plasmid expressing HIV-1 Vpu, a known BST2 antagonist (Fig. 1G).

### 3.2 hBST2 does not affect the early stages of the SBV replication cycle nor the intracellular localization of its nucleocapsid protein

We infected 293-hBST2 and 293-control cells with SBV to address whether hBST2 impacts the early stages of the SBV replication cycle. We then counted the number of infected cells at 8 and 12 h post-infection by immunofluorescence using an antiserum against the SBV N protein. We reasoned that if hBST2 blocks SBV entry, we would find a reduction in the number of virus positive cells early after infection. We found no difference in the number of SBV positive cells between 293-hBST2 and control cells indicating that hBST2 does not impact SBV entry (Fig. S1A). In addition, we found no difference in the intracellular distribution of SBV nucleocapsid (N) protein between 293-hBST2 and 293-control infected cells by confocal microscopy (Fig. S1B).

**Fig. 2:**
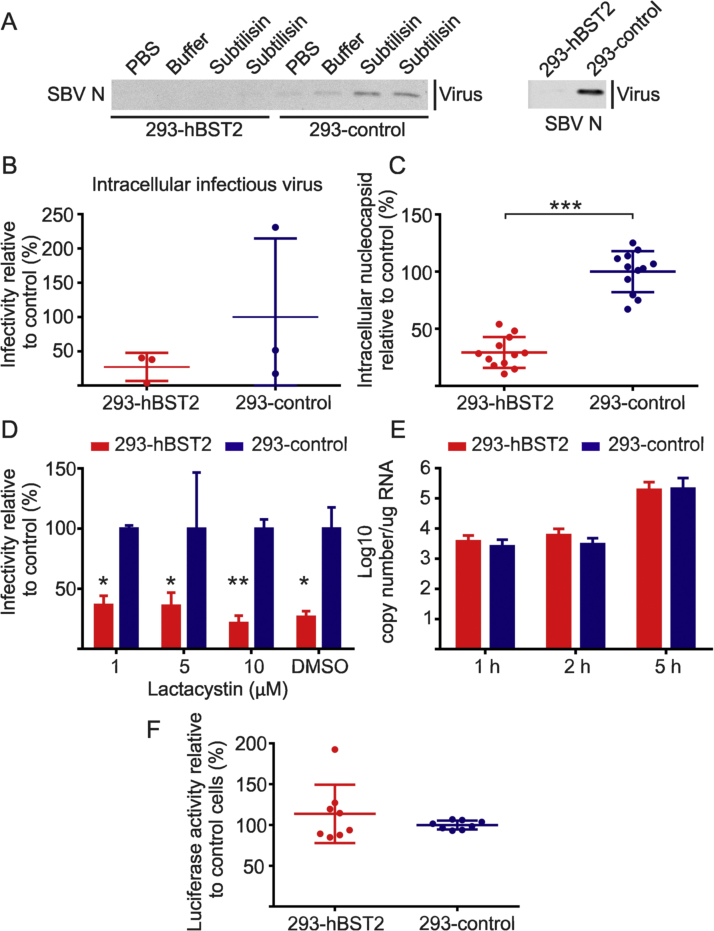
**hBST2 does not induce the accumulation of SBV within the cytoplasm or cell membrane of infected cells.** (A) 293-hBST2 and control cells were infected at a MOI of 0.001 and supernatants collected after 48 h. Cells were then incubated with PBS, buffer or subtilisin A (in duplicate) and supernatants were collected, filtered and virions pelleted by centrifugation. The right panel shows SBV virions recovered from supernatants before treatment to show hBST2 restriction of SBV which was complete in this case. The experiment was repeated independently three times. (B) 293-hBST2 and 293-control cells were infected at a MOI of 0.001 and 48 h post infection virions were released from the cytoplasm by freeze and thaw cycles. Infectious viral titers were determined by limiting dilution. The graph shows the average and standard deviation of three independent experiments. (C) 293-hBST2 and 293-control cells were infected at a MOI of 0.001 and 48 h post infection cells were lysed and analyzed by quantitative western blotting using a SBV N antibody. γ-tubulin was used to equilibrate protein loading. Results are presented relative to control cells. The graph shows the average and standard deviation of three independent experiments (*t*-test p < 0.0001). (D) 293-hBST2 and 293-control cells were infected with SBV at a MOI of 0.001. 5 h post infection the culture media was supplemented with 1, 5 and 10 µM of lactacystin. Cell lysates were collected 14 h later and analyzed by western blotting as described in C. Results show the average of two independent experiments performed in triplicates and are presented relative to control cells (unpaired *t*-test). (E) Quantification of viral mRNA (S segment) by qRT-PCR in 293-hBST2 cells and control cells. Values represent the average of three independent experiments and are presented relative to control cells (unpaired *t*-test). (F) SBV mini replicon assay in 293-hBST2 cells and 293-control cells. Values represent the average of four independent experiments and are presented relative to control cells (100%; unpaired *t*-test). *P ≤ 0.05; **P ≤ 0.01; ***P ≤ 0.001; ****P ≤ 0.0001.

There was no evidence of accumulation of SBV N at the cell membrane of 293-hBST2 infected cells by confocal microscopy, a typical phenotype observed as a result of BST-2 restriction (Blondeau et al., 2013, Neil et al., 2008, Wang et al., 2014). In order to confirm these data, we performed a protein stripping assay. If hBST2 causes protein tethering on the surface of virus-infected cells we would expect to recover more virions from hBST2-expressing cells than control cells after protease treatment. 293-hBST2 and control cells were infected with SBV and 48 h post-infection virions were stripped from the cell surface by treatment with the protease subtilisin-A. Controls included treatment with PBS and the buffer used for subtilisin-A reconstitution. Interestingly, more virions were recovered from control cells than from hBST2- expressing cells (Fig. 2A).

We then quantified and compared the amount of intracellular infectious virus in 293-hBST2 and 293-control cells. Cells were infected with SBV and after 48 h supernatants were removed and cells lysed by freeze-thawing. Infectious titers of cell-associated SBV particles were measured by endpoint dilution analysis. We found the titers of cell-associated SBV preparations derived from 293-hBST2 lower compared to control cells in each of three independent experiments, although this difference did not reach statistical significance (Fig. 2B). Hence, we also assessed the relative amount of intracellular SBV N in lysates of infected cells by quantitative western blotting. We found a significant reduction in the amount of SBV N in lysates derived from 293-hBST2 compared to control cells (Fig. 2C). To determine if hBST2 induces the degradation of SBV proteins, we repeated the experiments above in the presence or absence of the proteasome inhibitor lactacystin. Lactacystin treatment could not rescue the decrease of SBV N in cell lysates indicating that hBST2 does not induced the degradation of SBV virions via the proteasome (Fig. 2D).

Taken together these results indicate that both the steady-state levels of cell-associated SBV proteins and virions released in the supernatants are reduced in the presence of hBST2. Therefore, we investigated if hBST2 has an impact in the activity of SBV polymerase and/or the formation of the ribonucleoprotein complex (RNP). To this end, 293-hBST2 and 293-control were infected and viral RNA quantified at 1, 2 and 5 h post-infection by qRT-PCR. We found no statistically significant differences in the amount of viral RNAs in cells expressing hBST2 compared to control cells, indicating that hBST2 has no impact on SBV polymerase activity (paired *t*-test) (Fig. 2E). In addition, we used a mini replicon assay for SBV in order to assess viral polymerase activity in either 293-hBST2 and 293-control cells as previously described (Dong et al., 2013). We found no differences in luciferase activity between cells expressing hBST2 and control cells indicating that hBST2 does not impact SBV polymerase activity or the formation of the RNP complex (Fig. 2F).

### 3.3 hBST2 restricts SBV replication by reducing the amount of envelope glycoproteins in viral particles

**Fig. 3:**
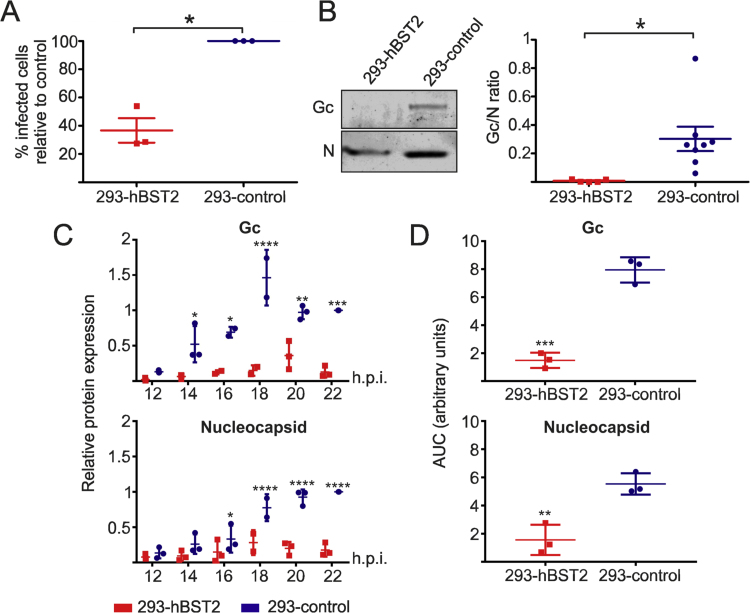
**SBV virions produced in 293-hBST2 are less infectious due to reduced levels of envelope glycoprotein.** (A) 293-hBST2 or 293-control cells were infected with SBV (MOI 0.001) and cultured for 48 h when supernatants were collected, filtered through a 0.45 µm filter and the number of SBV genomes quantified by qRT-PCR. CPT-Tert cells were then infected with 2.5 × 10^5^ SBV genome equivalents of these supernatants for one hours at 4 °C to synchronize infection followed by 1.5 h at 37 °C. 8 h post infection cells were fixed and stained by immunofluorescence using an antiserum against SBV N. The number of SBV infected cells was counted (10 fields of view per condition) and compared between groups. Three independent experiments were carried out using three independent viral preparations produced in 293-hBST2 and 293-control cells. Values represent the average of three independent experiments and are presented relative to control cells (100%) (*t*-test p < 0.05). (B) SBV stocks were produced as described in A and virions pelleted through a 20% sucrose cushion by centrifugation followed by quantitative western blot analysis. The graph displays the average of the Gc/N ratio of at least five independent experiments (*t*-test p < 0.05). (C) 293-hBST2 or 293-control cells were infected with SBV (MOI 0.001) and total cell lysates were collected at the indicated times post infection and analyzed by quantitative western blotting against SBV N and Gc proteins. The data is expressed relative to the expression of control cells at 22 h post-infection. The graph displays the average of three independent experiments (two-way ANOVA). (D) The area under the curve (AUC) for each of the individual experiments depicted in (C) was estimated and compared between groups (*t*-test). *P ≤ 0.05; **P ≤ 0.01; ***P ≤ 0.001; ****P ≤ 0.0001.

Next, we compared the infectivity of SBV virions released into the supernatant of cells expressing hBST2 or control cells. 293-hBST2 and 293-control cells were infected and 48 h later supernatants collected, filtered and the number of viral genomes quantified by qRT-PCR. These supernatants were then used to infect CPT-Tert cells (sheep choroid plexus cells) using 2.5 × 10^5^ of SBV genome equivalents. 8 h post-infection CPT-Tert cells were fixed and analyzed by confocal microscopy using an antibody against SBV N. The number of SBV positive cells was counted and compared between groups. We found a significant decrease in the number of SBV positive cells in CPT-Tert cells infected with 2.5 × 10^5^ genome equivalents of SBV generated in 293-hBST2 compared to those infected with the equivalent amount of genomes generated in 293-control cells (Fig. 3A).

Next, we assessed the relative amount of the Gc SBV glycoprotein present in SBV virions generated in 293-hBST2 and 293-control cells. Virions in supernatants collected from infected cells were pelleted and analyzed by western blot analysis. SBV N and Gc were quantified and the Gc/N ratio calculated (Fig. 3B). We found a significant decrease in the Gc/N ratio in SBV virions produced in 293-hBST2 suggesting that viral envelope protein incorporation is hampered by hBST2. In order to understand whether the reduced amount of Gc glycoprotein in released virions was the result of a reduced production of Gc in cells stably expressing hBST2 or rather due to the reduced incorporation of Gc into nascent virions, we quantified the amount of Gc in total cells lysates of 293-hBST2 and 293-control cells infected with SBV. To capture the kinetics of Gc expression. To capture the kinetics of Gc expression, we collected lysates early post infection starting from 12 h, which was the earliest time point in which we could detect viral protein expression by western blotting (Fig. 3C). We found statistically significant reduced quantities of Gc glycoprotein in hBST2 cells relative to control cells at all times post infection except at 12 h. At the same time, we quantified the quantities of N and we found that these were also reduced in SBV-infected 293-hBST2 relative to 293-control cells, but the differences reached statistical significance only at 16 h post infection. We also quantified the relative effect of hBST2 on Gc and N expression by estimating the area under the curve (AUC) between 12 and 22 h post infection in 293-control and 293-hBST32. We found a bigger difference in the AUC between 293-hBST2 and 293-control cells for Gc than for N, supporting the idea that hBST2 targets Gc production more effectively.

### 3.4 BST-2 orthologues restrict orthobunyaviruses in a host-dependent manner

**Fig. 4:**
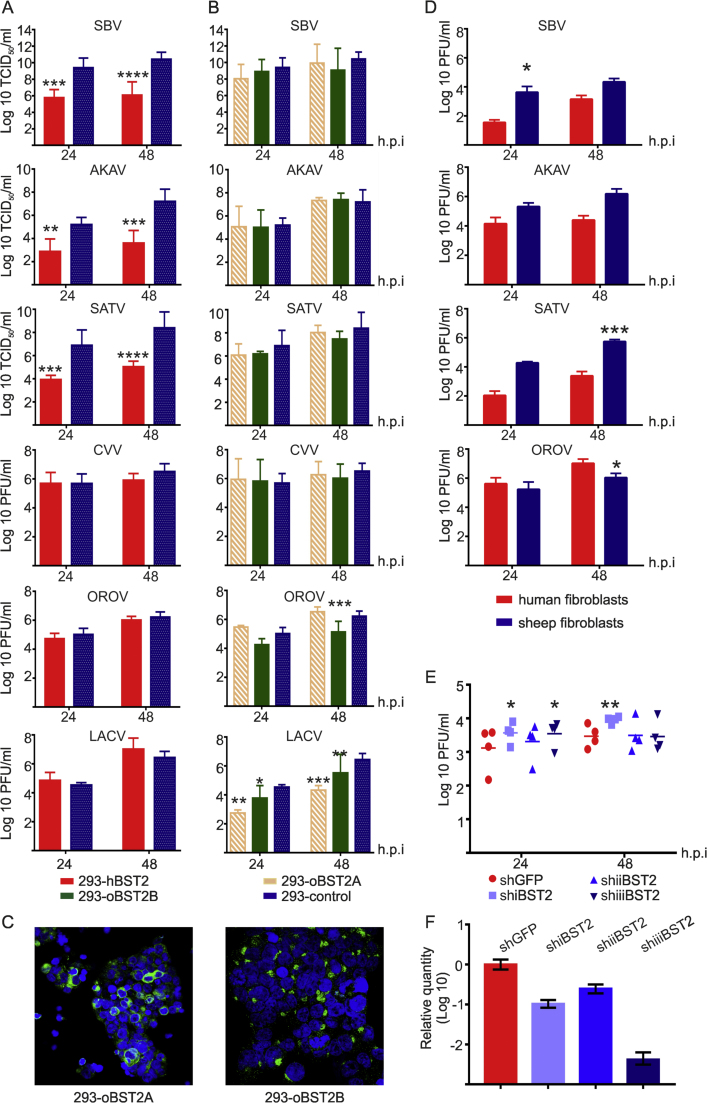
**Differential restriction of orthobunuyaviruses by BST-2.** SBV, AKAV, SATV, CVV, OROV and LACV growth kinetics in 293-hBST2 (A) and in 293-oBST2A and 293-oBST2B cells (B). Cells were infected at a MOI of 0.001 and virus growth monitored for 48 h. The graphs represent the average of three independent experiments using two independent virus preparations. Data was analyzed using a 2-way ANOVA. (C) 293-oBST2A and 293-oBST2B cells were analyzed by confocal microscopy using antibodies against the HA tag to detect BST2 (green) to confirm expression. (D) Differential growth of orthobunuyaviruses in sheep and human primary fibroblasts. Cells were infected with the indicated viruses at a MOI of 0.01 and virus replication monitored for 48 h. Data was analyzed using a 2-way ANOVA. (E) Human primary fibroblasts were transduced with lentivirus to stably express three shRNA against hBST2 (shRNAi, shRNAii and shRNAiii) or GFP as a control. Cells were infected with SBV at a MOI of 0.01 and virus titers assessed at 24 and 48 h post-infection. The graph represents the average and standard deviation of four independent experiments. Data was analyzed using a 2-way ANOVA. (F) Relative quantification of BST-2 transcripts in human primary fibroblasts stably expressing shRNAs against hBST-2 or GFP that was used as calibrator. *P ≤ 0.05; **P ≤ 0.01; ***P ≤ 0.001; ****P ≤ 0.0001.

We then monitored the replication kinetics of other ruminant orthobunyaviruses including AKAV and SATV in 293-hBST2 and 293-control cells. We found that hBST2 restricts the replication of AKAV and SATV (approximately 100 fold at 48 hpi), similarly to what we showed for SBV (Fig. 4A). On the other hand, hBST2 is unable to restrict the replication of OROV and LACV, both human orthobunyavirus (Fig. 4A) nor Cache Valley virus (CVV) an orthobunyavirus known to infect both ruminants and humans.

Given that BST-2 might have played a fundamental role in the transmission of pandemic HIV-1 M group from chimpanzees to humans (Sauter et al., 2009) and, as shown above, orthobunyaviruses with ruminant tropism are restricted by hBST2 while orthobunyaviruses with human tropism are not, we asked the question as to whether BST-2 restriction correlates with orthobunyavirus host range. To this end, we developed HEK 293T cell lines stably expressing the ovine orthologues of BST-2 (oBST2A and oBST2B) and assessed the replication kinetics of the bunyaviruses used above. We found that the replication of orthobunyaviruses with only ruminant tropism (SBV, AKAV and SATV) was not impaired by oBST2A and oBST2B (Fig. 4B). However, the replication of human-tropic OROV and LACV was restricted by the ovine BST2 orthologues (Fig. 4B). As expected the ovine orthologues could not restrict the replication of CVV. The patterns of expression of the 293-oBST2A and 293-oBST2B cell lines are shown in Fig. 4C. Altogether, these data suggest that there is a correlation between the capacity of BST2 to restrict orthobunyavirus replication and their host range.

To corroborate our results in a more relevant in vitro model, we assessed the replication kinetics of some of the viruses described above in primary sheep and human fibroblasts. We found species-specific restriction of virus replication: all ruminant viruses (AKAV, SATV and SBV) replicated to higher titers in sheep primary fibroblasts compared to human fibroblasts, while OROV replicated to higher titers in human fibroblasts compared to sheep fibroblast (Fig. 4C). Moreover, the replication of SBV in human fibroblasts in general increased when cells were transduced to stably express shRNAs against hBST2 (Fig. 4E). The levels of BST-2 knockdown are shown in Fig. 4F.

**Fig. 5:**
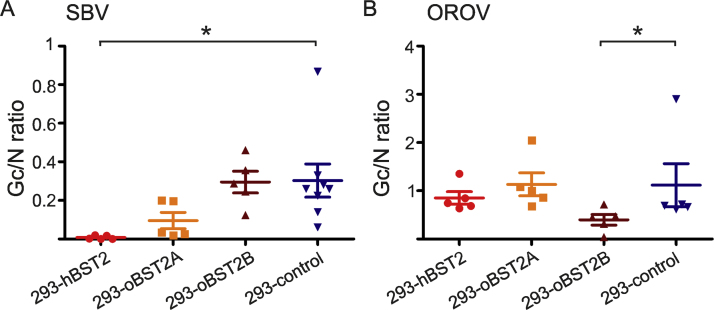
BST-2 restricts bunyavirus replication in a host specific manner by modulating the incorporation of envelope glycoprotein into virions. SBV (A) and OROV (B) stocks were produced in the indicated BST2-expressing cells and virions pelleted by centrifugation followed by quantitative western blot analysis. The graph displays the average of the Gc/N ratio. The data of the Gc/N ratio of SBV in 293-hBST2 of Fig. 4B has been included here for comparative purposes. Data was analyzed using a 1-way repeated measures ANOVA followed by Dunnett's multiple comparison test. *P ≤ 0.05; **P ≤ 0.01; ***P ≤ 0.001; ****P ≤ 0.0001.

We then examined the ability of the human and ovine BST-2 orthologues to modulate the incorporation of envelope glycoprotein into SBV and OROV virions. SBV and OROV stocks were produced in 293-hBST2, 293T-oBST2A, 293T-oBST2B or 293-control cells. Virions in the supernatants were pelleted followed by western blot analysis. SBV and OROV N and Gc were quantified and the Gc/N ratio calculated (Fig. 5). As expected, we only found a reduced Gc/N ratio for SBV when produced in hBST2 expressing cells but not when produced in oBST2A and oBST2B cells (p = 0.0147). Conversely, we found a reduced Gc/N ratio for OROV only when stocks were produced in oBST2B cells, the only cell line able to restrict OROV replication (p < 0.05). We observed a substantial difference in the Gn/N ratio between SBV and OROV, however we attribute this difference to the differences in sensitivities of each of the antibodies used against each viral protein.

## 4 Discussion

This is the first study identifying a host determinant of species susceptibility to bunyavirus infection. We showed that orthologues of BST-2, a cellular restriction factor with a broad antiviral activity against enveloped viruses, restrict orthobunyavirus replication in a host-specific manner. We found a correlation between the ability of orthobunyaviruses to replicate in the presence of different BST-2 orthologues in vitro and the range of their susceptible hosts in natural infections. In stable cell lines expressing different BST-2 orthologues, OROV and LACV replication was restricted by sheep BST-2 but not by human BST-2. On the other hand, viruses with ruminant tropism, such as SBV, SATV and AKAV, were restricted by human BST-2 but not by the ruminant orthologues. In addition, CVV, a virus of both humans and ruminants, was not restricted by either orthologue. We found delayed replication of the human virus OROV in primary sheep fibroblast compared to primary human fibroblasts while the converse was observed with the ruminant orthobunyaviruses SBV, SATV and AKAV. Moreover, knock down of human BST-2 by small hairpin RNA facilitated replication of SBV in primary human fibroblasts.

It is important to stress that orthobunyaviruses encode a non-structural protein (NSs) that inhibits global cellular transcription and therefore the activation of ISGs in general (Elliott, 2014). Hence, it may be counterintuitive to expect that individual ISGs may contribute to determine the host range of orthobunyaviruses considering that these viruses hamper the host antiviral responses by blocking expression of all ISGs. However, some restriction factors such as BST-2 are constitutively expressed in some cell types in addition to being expressed in response to IFN production. These factors are often referred to as part of the “intrinsic” immune response of the host as they are available in the cell even before pathogens are sensed and the IFN response is initiated (Yan and Chen, 2012). Here, we show that BST-2 restricts orthobunyavirus replication in a species-specific manner. In vitro, orthobunyaviruses are able to infect a variety of cell lines of different species. This indicates that the host range of these viruses does not totally depend on receptor availability or other factors required for viral replication. For example, SBV antibodies have been detected in deer, elks, buffalos, alpacas and dogs among others (Lievaart-Peterson et al., 2015) but so far disease has only been documented in ruminants in the form of abortions and congenital malformations. AKAV has been detected in camels, horses, buffalos and dogs but it is only pathogenic in domestic ruminants (Kirkland, 2015). OROV causes febrile illness in humans but it has also been isolated from primates and sloths (Bastos Mde et al., 2012) while LACV causes encephalitis in humans but no disease presents in chipmunks or squirrels which are also hosts (Beaty and Calisher, 1991, Grimstad, 1988). We found that human BST-2 was unable to restrict replication of OROV and LACV while the ovine orthologues did not restrict the growth of the ruminant viruses SBV, AKAV and SATV. Interestingly, neither the human nor the ruminant BST-2 impaired the replication of CVV which has been associated with abortions and congenital malformations in ruminants as well as encephalitis in humans (Chung et al., 1990, Sexton et al., 1997). These results are in accordance with a previous report which showed that human BST-2 does not restrict the replication of Rift Valley Fever virus, a phlebovirus responsible of congenital abnormalities in ruminants as well as the cause of febrile illness in humans (Radoshitzky et al., 2010).

Under our experimental design we showed that BST-2 does not completely abolish viral replication, in line with the activity of this protein against other enveloped viruses and thus BST-2 proteins of different species likely restrict orthobunyaviruses with different efficiency. However, the role of BST-2 in influencing orthobunyavirus host range will likely derive from a variety of factors including its sites and timing of expression and its efficiency of restriction of a given virus. Ultimately many other factors, including the timing and efficiency of total cellular protein shut-down induced by viral NSs will determine orthobunyaviruses host range.

BST-2 is a versatile molecule that is not only able to restrict replication of a variety of enveloped viruses but it is also implicated in innate sensing, signaling and structural organization of the cell (Sauter, 2014). The most common mechanism of viral restriction by BST-2 described so far involves the physical attachment of virions to the cell membrane of infected cells (Perez-Caballero et al., 2009). However, we previously showed that the ovine orthologue oBST2B restricts sheep retroviruses by reducing the incorporation of envelope glycoprotein into nascent viral particles (Murphy et al., 2014). Here we found that hBST2 restricts SBV replication by reducing the incorporation of the Gc glycoprotein into virions.

The mechanisms by which BST-2 targets orthobunyavirus glycoproteins are not known. However, it does not seem to be the result of a general disruption of viral protein synthesis or turnover by BST-2 given that the same orthologue expressed in the same cell line has a different impact on replication of different orthobunyaviruses. In addition, restriction is not impacted by the addition of inhibitors of the proteasome thus discarding the possibility that BST-2 can induce the degradation of viral proteins. One of the ovine paralogues (oBST2B) lacks a predicted glycosylation sites and the carboxy-terminal GPI anchor which results in a protein that is retained within the Golgi apparatus. Bunyaviruses are thought to assemble at membranes of the Golgi apparatus that have been modified by the insertion of the viral glycoproteins. Newly formed viral RNPs locate beneath the modified membranes. It is believed that the interaction between the RNPs and the cytoplasmic tail of either or both the viral glycoproteins triggers budding (Overby et al., 2007, Shi et al., 2007). Thus, crosstalk between orthobunyavirus glycoproteins and BST-2 orthologues may occur in this cellular compartment. However, we found no SBV Gc and hBST2 colocalization in infected cells or expressing SBV Gc by transient transfection indicating that the mechanism of action of hBST2 might be either indirect or involve interaction with another viral protein (i.e. Gn). On the other hand, we cannot assume that glycoproteins from different orthobunyaviruses are translated and traffic in the cytoplasm in exactly the same way. Hence, the species-specificity of BST-2 restriction could be related to differential translation and recruitment of viral proteins for budding.

Emerging infectious diseases pose a threat to human and animal populations. Viral emergence is a multifactorial event where ecological, genetic, immunological and evolutionary processes play a key role in determining its outcome. In general, little is known about the adaptive changes that viruses require to cross the species barrier and emerge into new species (Holmes and Drummond, 2007). Until now, scarce information existed on the host genetic factors that determine orthobunyavirus host range and what rules the emergence of these pathogens. This study provides for the first time clear clues on one of the host genetic barriers that these viruses face to establish themselves in a new host species.

## Footnotes

Appendix A Supplementary data associated with this article can be found in the online version at doi:10.1016/j.virol.2017.06.017.
