# Supplementary material for: Sensitivity to BST-2 restriction correlates with Orthobunyavirus host range
Source: Virology. 2017 Sep;509:121–30. doi: 10.1016/j.virol.2017.06.017 (PMC5526858; doi:10.1016/j.virol.2017.06.017)
Supplement: Supplementary file 1 — Supplementary material [file mmc1.docx]

Figure S1


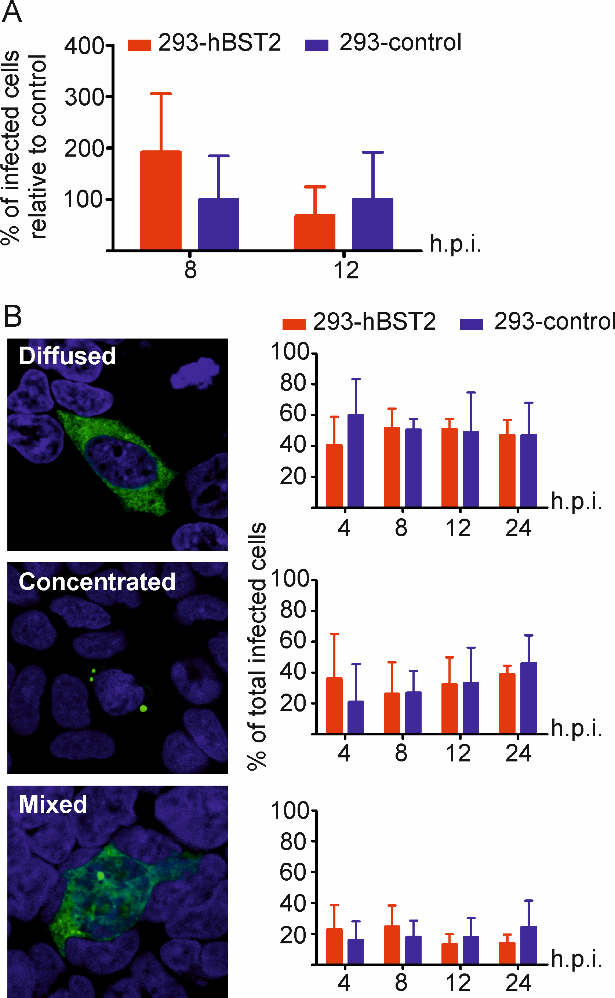


**Supplemental Figure S1. huBST2 does not affect SBV entry nor viral intracellular distribution.** A. 293T -hBST2 or 293T-control cells were infected with SBV (MOI 0.001) and 8 or 12 h post infection cells were fixed and processed for confocal microscopy using antisera against the SBV N protein. The number of SBV positive and negative cells was then counted in ten random fields. The graph shows the average and standard deviation of four independent experiments relative to 293T-control cells. B. 293T-hBST2 or 293T-control cells were infected with SBV (MOI 0.001) and fixed at four, eight, 12 and 24 h post infection followed by immunofluorescence and confocal microscopy using antisera against the SBV N protein. The number of cells displaying three different patterns of SBV N distribution: diffused, concentrated and mixed was counted (nine to 19 random fields per condition) and the percentage of each of them in relation to the total number of infected cells was calculated and compared between cell lines. The graph shows the average and standard deviation of four independent experiments.
